# East and West

**Published:** 1887-11-26

**Authors:** Leslie Keith

**Affiliations:** Author of "The Chilcotes," "Uncle Bob's Niece," etc.


### East and West.

By Leslie Keith.

Author of "The Chilcotes," "Uncle Bob's Niece," etc.

(*Continued from p.* 138.)

Chapter IX.—Facing the Question.

Alicia did not fail to return as she had promised. She went this time in an omnibus, and thence on foot to Baron Street, and she wore a dress that was severely restricted in ornament.

If she hoped by these concessions to identify herself with the new world, she signally failed, however, and her plain dress only served to emphasise her natural grace and dignity. She was of another order, and David Jardine, glancing up from his papers, looked at her dreamily, as if she were the embodiment of one of his fancies who had taken earthly shape. Alicia looked at him in turn with a surprise she could not veil. Seated in Mary's place by the open window was a slender youth; narrow-chested, hollow-cheeked, a dweller in the "glooming alleys" all his days, one would have said, rather than a son of the hills. The sweet breath of the country might have kept the life in him, but the fevered air of London was killing him. It needed no experienced eyes to see that he was dying—slowly, perhaps, but inevitably. Alicia met the gaze of the luminous brown eyes with something between pity and shrinking in her own: to die; to give up the struggle; to be swept away by fate with scarce a resistance: her own splendid vitality revolted with a kind of fierceness from this yielding.

"Are you David Jardine?" she asked, advancing a step. A farmer this; a shepherd? She could not adjust the imagined picture with the real.

"Yes," he said. "You are Miss Frankland, who came to see Mary yesterday. She told me—and you left this." His eyes wandered from her face to the table, where, among his scattered papers, there stood, in a cupful of water, the rose she had worn.

"I used to think little enough of flowers once," he said, with a smile that explained everything. A rose had, no doubt, been a useful poetical property then, and nothing more, as was the yellow primrose to the worthy Peter Bell.

"I will bring you some to-morrow," said Alicia, accepting the chair he offered. "Where is Mary?"

David looked round with a frown of sudden annoyance, as if he had not missed her till then.

"She has gone out, I suppose," he said. "I don't know why she goes—and always just when one wants her."

Alicia could guess why she went—to bring some more needlework home, no doubt, or to buy the dinner David ate without troubling himself to learn how it was paid for.

"She will come back presently. You will wait?"

"I have come to spend the day. While we wait for her will you read me some of your verses?"

A poet seldom requires much pressing to yield to such an invitation. He believes in his own divine fire; he could not else be a poet. It was this consciousness, this inner sense of power, that sustained David and lifted him from despair; that blinded him to the misery around him, and to his own growing share in it. Happy he who can enter at will into this region of pure fancy, where sorrow and disappointment and heartburning do not follow him. Nevertheless, however secure one's self-belief, it is very precious to make a convert, and David was consequently a little fastidious in his selection.

Alicia folded her hands and prepared to listen. She had gauged the young man's weakness and vanity already, and her mental bent, as we know, was towards criticism. Yet while he read, almost against her will she was led to share his confidence. A sympathetic voice and a perfect knowledge of his own strong points may have helped this, and it was only when he faltered and ceased that her stronger judgment prevailed. She had a tolerably cultured taste, having found time even in a determinedly idle life for some study; and she knew that the poet was no genius. The verses were smooth and sweet, with a hint of that morbid sadness in them that youthful versifiers love; they had facility and ease, but these graces are shared by many, and it takes something more to catch the world's dull ear.

"You came to London to find a publisher?" she asked.

"Yes," he said. He hung his head, and then he lifted it again. "I will find one," he said almost defiantly. "It is a mere matter of time—of gaining a hearing. Almost everyone suffers rejection at first; many works destined to become famous have had their season of refusal. I mean to succeed."

Alicia looked at him very gravely—at his eager eyes, his thin face, and shadowy hands. Not the hands these of a son of the soil. If success were to come, it had need to come quickly.

"I think you will succeed," she said, and while she spoke she mentally registered a resolve.

It was upon this scene that Mary entered. David's face was brighter than it had been for a long time. The vanity that was so lightly stirred had had many a wound and rebuff; the interest and encouragement of a gracious and cultured woman of the world was as balm and healing. But it was the sister's gratitude that Alicia had earned; to cheer David, to raise his drooping spirits, and soothe his sensitive nerves—these were the services that claimed her lasting love. When the young man had tied up his papers and gone out, impelled by the restlessness that haunted him, Alicia had but to propose for Mary to acquiesce.

"Mary," she said, "put away your sewing. If it must be done, my maid shall do it. She may as well hurt her fingers as you. I want you to go out with me. I want you to show me the homes of the people here. I wish to understand how they live. But remember, I am not going as a philanthropist; I have brought neither tracts nor money to distribute."

"A kind word will go farther than either. It is not the giving of money that will help," said Mary, as she folded her work.

"It is the only kind of help we recognise. It is not very much trouble; so we give our silver or our gold, and think we have done our whole duty. What does it matter to us how it is spent, or who benefits? That is not our affair."

"Come and see," said Mary.

That was the first of a long series of explorations with which Alicia Frankland employed the leisure of the summer days. It began with a few homes where Mary had made herself welcome by little services of love, and it gradually embraced a wider area. Alicia had no innate taste for the work of ministration; she had kept herself aloof from all such womanly labours. She went as an observer, a spectator, rather than an actor; but it was with the same almost fierce earnestness with which she had taken her amusements that she now faced the social problems which were only then beginning to press themselves upon public attention. Slumming was as yet an undreamed-of pastime among the fashionable; class still stood aloof from class, and the western half of London knew and cared nothing for the doings and sufferings of the east. It turns away from this unknown land of set purpose and on principle. Knowledge would bring discomfort, and even a well-tutored conscience might rebel before the evidence of the eyes; therefore it is our way to summon a passable philosophy—to shrug our shoulders—to go by on the other side. We cannot help; the wound is byond our tinkering; why, therefore, needlessly harrow our feelings?

There was, of course, a strictly-drawn limit to the adventures on which our pilgrims were bent. There are many places in sad London where girls cannot venture alone, though these are fewer than they were; but even in the courts and streets where an Alicia and a Mary may penetrate without fear of insult, enough may be seen and understood to awaken thought. Alicia brought to the study of this phase of London life an absolute ignorance that is more rare in the days that have converted the poor into a fashionable show, and made their sufferings the favourite theme of sensational pens. We read of life in cellars and garrets, in rotten, reeking tenements; of the one-roomed houses where families herd like wild beasts, and live on garbage that our dogs would reject; of lives that are one sustained struggle, one long defeat; of manners and morals as pestilential as the sewage gases that poison the air: we read of them till our senses are blunted, our hearts grown hard; till, staled by frequence, we listen as to an oft-told tale which has lost its power to touch us. For this apathy that clogs the wheels of progress there is but one remedy; we must put away the books and pamphlets, and go down among those brothers and sisters for whom, as for us, Christ agonised, and share, if but for a moment, the bond of simple human fellowship. To see with the eyes, to touch with the hand those other toil-worn hands, to breathe, even passingly, the anguish and the smart, and suffer the stress of battle—who is there that can know this experience, and turn away once more, saying, "I have no part or lot with these?"

The determining acts of our life have often very small beginnings. A whim, a pique, a restless craving to be taken out of herself, had led Alicia's steps towards Bethnal Green, and here she was caught, bound, held fast. In the course of her study she developed a curious passion for statistics; she posed her simple companion with questions her inexperience could not answer. Mary's heart had been touched, but Alicia's intellect demanded to be satisfied. Therefore the accumulated questions were all poured out on Felix Mervyn the first time that young man recrossed her path.

This did not happen for a week or two, because the young doctor, eagerly bent on his profession and interested in its scientific aspects, found his time largely absorbed by its demands, and had little to spare for a young lady who might, for all he knew, be merely playing at philanthropy as he and she together had played at so much in life. Yet doubtless he was not without acquaintance with her doings, and possibly knew more of them than she dreamed. He was not thus wholly unprepared for her questions, and was quite ready, when at last they met, to answer them.

"Why do the poor crowd together?" she asked, as everyone naturally asks to begin with. "It is not that they love each other; their family affection can scarcely demand that eight of them shall live, eat, sleep, wash, in a room eight feet square."

"The Artisans' Dwellings Act has something to answer for. It has swept away the old homes, and by halving the accommodation has doubled the population of each house. You have it to thank for the extortionate rents that are still eagerly paid for those vile dens you have read of. It has ground down one-half of the poor to help the other half, and enrich unscrupulous landlords."

"But why can't the people who have been made homeless, as you say, by these improvements, move into the new buildings? Surely they don't prefer the old?"

"Because they are not wanted there. There isn't room for them; and if there were, they couldn't afford the rent. Besides, their manners and tone fall below the desired standard."

"I don't understand."

Mervyn laughed. "There are grades even here," he said. "You don't perceive the difference, perhaps, but it exists. The inhabitants of the model dwellings are the aristocracy of the poor. To qualify for rooms there a man must be married, his children vaccinated; he must be in receipt of a wage that will guarantee his ability to pay his rent weekly; and, moreover, he must not be engaged in any occupation that will make him an offence to his neighbours. These conditions do not seem overwhelmingly difficult to you and me, but when you consider what the people here are, and what unsavoury businesses many of them take to, you must see at once what a strict line these rules draw. They shut out the costermonger, the rabbit-puller, the mangle-woman—even the French polisher; in short, it's a filtering process that very few can survive."

"Why don't they go to the country, then?" said Alicia, with a frown. "The country is wide—there is room in it for everybody."

"Because," said Mervyn, suppressing a smile at this innocent solution of the difficulty, "we are talking of people who must work or starve, and whose work lies in the heart of London. You can't expect a man who is habitually underfed to tramp several miles morning and evening to and from his work, and out of a wage of twelve or fifteen shillings where are railway fares to come from? Even the women can't afford to run the risk of losing a chance of employment. Those who work for the slop tailors, the match-box makers, and the sack-makers, for instance, have got to take their work home; the factory girls must be punctual to a certain hour every morning; even the charwoman has her beat, and would starve if she left it for any such sentimental reason as fresher air and more elbow-room."

Alicia looked at him with sombre brows.

"Is there, then, no remedy?" she asked. "Must these things go on?"

Felix Mervyn shrugged his shoulders. "He will be a clever man who can answer that question satisfactorily," he said.

"And yet," she said, looking at him wonderingly, "you make it your mission to help these people to live."

Mervyn laughed as he prepared to go.

"They wouldn't thank me for shortening their term," he said. "Life is sweet, even here."

He thought, perhaps, a little more respectfully of his questioner as he went upon his rounds. He was not so very certain now that she was amusing herself. He remembered a certain gravity and concentration of purpose that she had brought even to her play in the days when he had shared her frivolity, and he saw it taking a new form here. And yet, what could come of it all? For a moment she might be impressed—moved to indignation; but she would go away again and forget it all. He knew very well how easy it is to forget. He belonged to another world from hers, a world in which he was a voluntary exile, and when she left it and returned to her own, he knew that he should see her no more. He suffered a vague ache at the thought.

Alicia, as will be seen, was facing the question as a mere tyro. Her difficulties were the difficulties of a beginner, but they were, after all, difficulties that have as yet proved unsolved problems to those who have most deeply pondered the matter.

But she also brought the earnestness of a beginner to her task. She brooded darkly over the things that she daily saw and heard, and found it difficult, as all young people do, not to believe in some sharp and swift remedy. It was also significant that she no longer talked of amusing herself or relieving the tedium of her days. The humour that Mervyn found sustaining in the course of his practice even seemed to her to have an edge of hardness in it. She had herself—being a woman—scarcely humour enough to perceive that unless he could lighten the tragedy with a jest, he had scarcely been able to support its burden.

When Kitty wrote enthusiastically of the delights of Homburg and of those Paris toilets that made her own share in them a triumph, Alicia tore the letter into very small pieces. The world of bands, of promenades, of flirtations, of drives and picnics and balls seemed as far off and unattainable as Paradise to the dwellers in the Inferno. Yet she had not so much as a passing wish to re-enter that Paradise where Kitty was exacting a diligent homage from her lover, and where Mr. Frankland found it possible to smoke even more cigars than in London.

Alicia came daily to Baron Street with the punctuality of a dock labourer in search of employment, and almost with the same eagerness. She returned nightly to Park Lane, being mindful of that promise her father had drawn from her; but with the autumn days her outsetting in the morning grew earlier, and her home-going in the evening as late as she dared to make it.

Her companionship was the one consolation of Mary's life, for the success which would have made her supremest joy had not come to David yet. He had found no wider audience than that composed of his sister and cousin; and indeed he was never likely to secure more patient listeners. If Alicia did not admire with the blindness of Mary, she found patience the easier, because she saw that the poet was bound for a country where the praise of men would no longer sound sweet in his ears.

Once only, when their comradeship had drawn them very near together, did she urge Mary to accept the help she would now so gladly have given, but Mary still refused.

"Not yet; we can manage yet," she said. "By-and-bye, perhaps, if David does not succeed just at once."

And in that answer Alicia discovered a pride as inflexible as her own, and a self-respect that she was compelled to recognise.

*(To be continued.)*

